# Epidemiological profile of *Neisseria meningitidis* in Casablanca, Morocco: 2010–2019

**DOI:** 10.1099/acmi.0.000157

**Published:** 2020-07-22

**Authors:** Khadija Ait Mouss, Aziza Razki, Eva Hong, Bahija Zaki, Fakhreddine Maaloum, Néhémie Nzoyikorera, Houria Belabbes, Naima Elmdaghri, Khalid Zerouali

**Affiliations:** ^1^​ Department of Microbiology, Faculty of Medicine and Pharmacy, Hassan II University of Casablanca, 19 rue Tarik Bnou Zyad, 20360, Casablanca, Morocco; ^2^​ Bacteriology-Virology and Hospital Hygiene Laboratory, University Hospital Centre Ibn Rochd, 1, Rue des Hôpitaux, 20100, Casablanca, Morocco; ^3^​ Institut Pasteur du Maroc, 1, place louis pasteur, 20360, Casablanca, Morocco; ^4^​ Institut Pasteur, Invasive Bacterial Infections Unit, Paris, France

**Keywords:** multilocus sequence typing, *Neisseria meningitidis*, nucleic acid amplification techniques, penicillin G

## Abstract

Surveillance of invasive meningococcal diseases (IMD) must be carried out regularly and continuously in order to detect the emergence of strains of reduced susceptibility to antibiotics for therapeutic and prophylactic use and the appearance of new invasive clones. Molecular-typing approaches allow reliable traceability and powerful epidemiological analysis. This is an epidemiological study of *Neisseria meningitidis *causing meningitis in Casablanca, Morocco. The grouping was confirmed by PCR mainly on the isolates from cerebrospinal fluid (CSF). A total of 245 confirmed isolates of *N .meningitidis* were obtained between 2010 and 2019 of which 93 % are of group B. Overall, 24 % of all the isolates have a reduced susceptibility to penicillin G, but no resistance to penicillin G has been reported. All the isolated strains are susceptible to third-generation cephalosporins (3GCs). Genotyping by multilocus sequence typing (MLST) of a selection of 18 strains showed that the majority of isolates belong to the invasive clonal complex CC 32(9/18) followed by the CC 41/44(3/18).

## Introduction

Invasive meningococcal disease (IMD) is a serious infection that occurs worldwide. *Neisseria meningitidis *remains one of the main causes of bacterial meningitis affecting all ages, mostly children. It can occur in sporadic cases form or causes epidemics as in ‘the sub-Saharan African meningitis belt’, which extends from Senegal in the west to Ethiopia in the east. The IMD rate is higher in that belt [1] with clonal epidemics due to the strains of serogroups A, W and recently serogroups X and C, which can cause epidemics in sub-Saharan Africa [2]. In Morocco, meningococcal disease is endemic-sporadic with an incidence rate ranging from 2 to 3.6 cases per 100 000 inhabitants [3]. In Morocco, IMDs are subject to compulsory declaration and represent in Morocco a public health problem. Meningococcemia can be the cause of death and have serious consequences. It requires emergency care and a regular epidemiological surveillance. A rapid and reliable etiological diagnosis as well as antibiotic susceptibility tests are therefore essential for the effective management of these infections. Beta-lactams are used in the treatment and prophylaxis of IMDs due to their high activity and their almost total absence of side effects [4]. On the other hand, third-generation cephalosporins (3GCs): ceftriaxone and cefotaxime remain the standard treatment when the germ is not identified and this from the age of 1 month [5]. Rifampicin is recommended for chemoprophylaxis.

*N. meningitidis* is a Gram-negative bacterium of the family *Neisseriaceae* [6] that infects humans only; it is transmitted directly from respiratory or salivary projections of patients and especially healthy carriers by prolonged and close contact, presenting itself as diplococcus in the direct examination of pathological products, the most often cerebrospinal fluid (CSF).

*N. meningitidis* is an encapsulated bacterium; its capsule is of a polysaccharide nature and its antigenic identity determines the serogroup. Six serogroups A, B, C, W, Y and X are responsible for almost all of the IMDs [7]. This pathologen *i*s naturally competent for transformation that allows a frequent recombination event between strains. These genetic events are responsible for the high genetic diversity of *N. meningitidis *. By applying molecular biology techniques such as multilocus sequence typing (MLST), it is possible to identify meningococcal clones responsible for major epidemics around the world and to elucidate their modes of transmission. A clone with epidemic potential can lead to worldwide spread in few years. Typing approaches allow reliable traceability of bacterial strains and powerful epidemiological analysis.

The main objective of this study is to describe the epidemiological profile of *N. meningitidis* in the Casablanca region (Morocco) over a period of 10 years since 2010. The secondary objectives are to assess the contribution of diagnosis by PCR when culture fails, to determine the distribution of serogroups, to monitor susceptibility antibiotics used in treatment and prophylaxis, and to analyse its genetic polymorphism.

## Methodology

### Characterization of isolates

It is a descriptive study on meningococcal strains isolated between January 2010 and December 2019 at the microbiology laboratory of Ibn Rochd University Hospital Centre (IR-UHC) of Casablanca and analysed in collaboration with the laboratory for surveillance of invasive meningococcal infections (IIM) at the Institut Pasteur in Morocco. The cultures were carried out on chocolate agar + Polyvitex (BioMérieux, Marcy l’Etoile, France) and incubated at 37 °C under 5 % CO_2_, while identification was carried out by standard bacteriology procedures. The MICs of penicillin G, ampicillin and ceftriaxone were determined by E-test (Oxoid Thermofisher, UK) on Mueller–Hinton agar in addition to 5 % of sheep blood (MHS) (BioMérieux, Marcy l’Etoile. France) and interpreted according to the Clinical and Laboratory Standards Institute (CLSI) 2016 recommendations [8]. Furthermore, susceptibility to rifampicin, ciprofloxacin and chloramphenicol was determined by the diffusion method [8].

For any negative culture after 48 h, multiplex PCR was performed on CSF targeting the genes: crgA, lytA and bexA to detect *N. meningitidis *, *Streptococcus neumoniae* and *Haemophilus influenzae,* respectively. CSF must meet the inclusion criteria; a number of leukocytes greater than 100/ mm^3^ with predominance of polynuclear neutrophils and a C-reactive protein (CRP) ≥50 mg l^−1^. The grouping of the isolates was confirmed by PCR [9].

Considering the dominance of serogroup B in IMDs in Casablanca and the high frequency of these infections in children under 1 year old, we selected the strains that met these criteria when performing MLST. The polymorphism analysis of *N. meningitidis* isolates by MLST was carried out at the National Reference Center (CNR) of Meningococci and Haemophilus of the Institut Pasteur in Paris (France), as described by Maiden and his colleagues [10] for seven housekeeping genes, namely the seven loci encoding the metabolic enzymes (abcZ, adk, aroE, fumC, gdh, pdhC, pgm) as well as PorA, PorB which are porins of the outer membrane, Fet A (transport protein iron) and Pen A (penicillin resistance gene). By combination of the alleles obtained for each locus, sequence type (ST) is defined and a clonal complex (CC) [11] is thus assigned. Primers, determination of sequence alleles and designation of sequence types are described on the MLST website (https://pubmlst.org/neisseria/).

## Results

### General characteristics of invasive meningococcal isolates

**Fig. 1.**
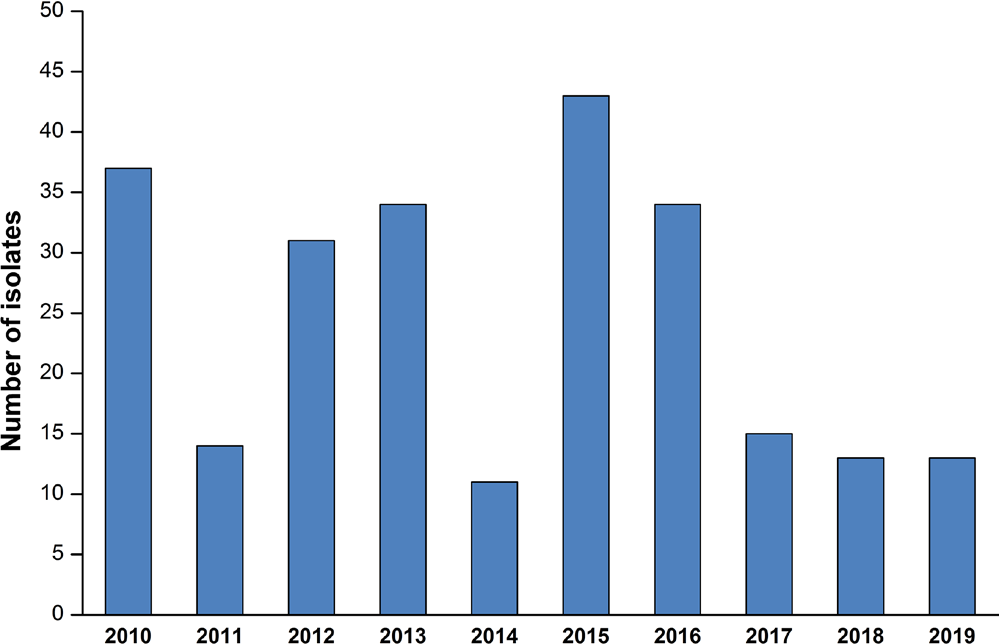
Annual distribution of IMD between 2010 and 2019 in Casablanca, Morocco (*n*=245).

It the period study, 245 cases of IMDs have been identified, including 183 cases (74.69%) of viable cultures and 62 cases (25.30%) identified by PCR. With regard to the isolation site, most cases were from CSF 75.10 % (*n*=184), 24.08 % (*n*=59) of cases were from blood culture while two strains were isolated from the articular liquids. The annual distribution of IMDs is heterogeneous; the largest number of cases was recorded in 2015 (Fig. 1). Meningococcal disease is more frequent during the winter–spring seasons with 32 and 33.5%, respectively. The most represented age group was children of 1 to 4 years old (35%), and 20 % of cases affected the age groups less than 1 year and 5 to 9 years, respectively. We noted a male predominance with 58 % of the cases, which gives a sex ratio of 1.37.

**Fig. 2.**
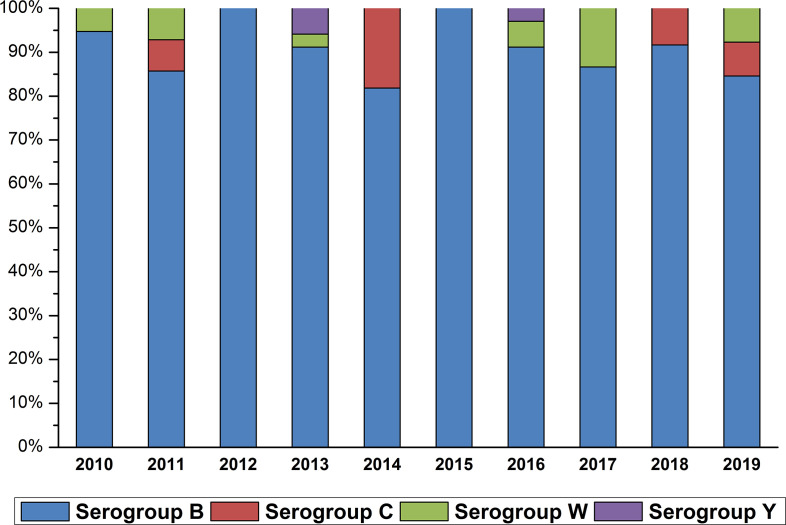
Annual distribution of serogroups (2010–2019).

In the Casablanca region, IMDs are mainly due to serogroup B with 93.06 % of cases, followed by serogroup W with 3.67 %. Only 1.22 % of the isolates are of serogroup Y and 2.04 % are of serogroup C. The distribution of serogroups over time is heterogeneous except in the years 2012 and 2015 where all IMDs were due to serogroup B (Fig. 2).

**Fig. 3.**
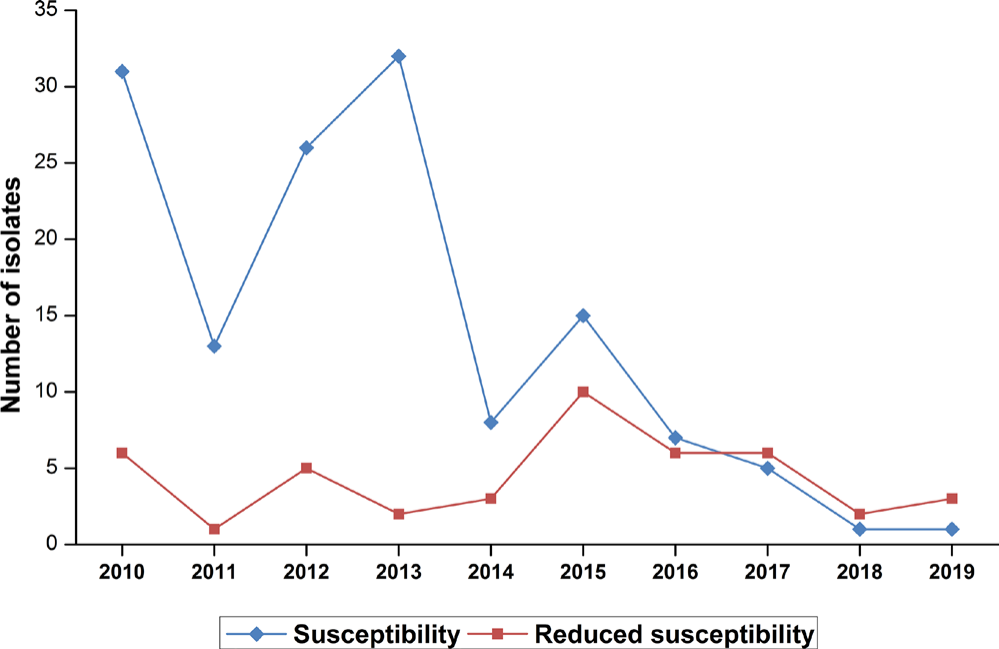
Reduced susceptibility rate of *N. meningitidis* isolates to penicillin G during the period from 2010 to 2019 (*n*=183).

Of the 183 strains isolated, 139 isolates (75.95%) are susceptible to penicillin G (MIC ≤0.06 µg ml^−1^) and 24.04 % (*n*=44) have reduced susceptibility (0.12≤MIC≤0.25 µg ml^−1^) (Fig. 3). *N. meningitidis* isolates of reduced susceptibility to penicillin G belong to 84.09 % to serogroup B (37/44), 9.09 % are of serogroup W (4/44), 4.54 % are from serogroup C (2/44) while 2.27 % are of serogroup Y (1/44). It should be noted that all the isolated strains are susceptible to 3GCs, ciprofloxacin and ampicillin. As for rifampicin, 33 strains (18.03%) are of reduced susceptibility. On the other hand, 7.65 % of strains (*n*=14) are of reduced susceptibility to chloramphenicol.

The molecular characterization by MLST (PorA, PorB, FetA and PenA) of 18 strains of meningococcus is summarized in Table 1. Analysis of the 14 serogroup B isolates revealed that the most common invasive clonal complexe was CC 32 (ST-33, ST-34 and ST-10792), followed by CC 41/44 (ST-1255, ST-6349). Two strains are part of CC 213 and CC 103.

**Table 1.** Genotypic characterization of the 18 *N. meningitidis* isolates

	MenB				MenC	MenW	MenY	
	CC 32	CC 41/44	CC 213	CC 103	CC 865	CC 11	CC 167	CC 174
**ST**	ST-33 (*n*=3)	ST-1255 (*n*=2)	ST-4224 (*n*=1)	nd (*n*=1)	ST-3327 (*n*=1)	ST-10791 (*n*=1)	ST1627 (*n*=1)	**ST 1466 (*n*=1**)
	ST-34 (*n*=2)	ST-6349 (*n*=1)						
	ST-10792 (*n*=1)							
	nd (*n*=3)							
**PorA-VR1**	P1.19 (*n*=7)	P1.7–2 (*n*=2)	P1.22 (*n*=1)	nd (*n*=1)	P 20 (*n*=1)	P 5 (*n*=1)	P 5–1 (*n*=1)	P 21 (*n*=1)
	P1.5 (*n*=1)	P1.22 (*n*=1)						
	nd (*n*=1)							
**PorA-VR2**	P15 (*n*=7)	P4 (*n*=2)	P 14 (*n*=1)	nd (*n*=1)	P 16–36 (*n*=1)	P 2 (*n*=1)	P 10–4 (*n*=1)	nd (*n*=1)
	P2 (*n*=1)	P 14–6 (*n*=1)						
	nd (*n*=1)							
**PorA-VR3**	P 36 (*n*=7)	P 32 (*n*=2)	P 36 (*n*=1)	nd (*n*=1)	P 37–1 (*n*=1)	P 36–2 (*n*=1)	P 36–2 (*n*=1)	P37–1 (*n*=1)
	nd (*n*=2)	P 36–2 (*n*=1)						
**PorB**	3–1 (*n*=8)	3–1 (*n*=2)	3–2 (*n*=1)	3–79 (*n*=1)	188 (*n*=1)	3–1 (*n*=1)	3–2 (*n*=1)	3–236 (*n*=1)
	3–343 (*n*=1)	339 (*n*=1)						
**FetA-VR**	F 5–7 (*n*=1)	F 5–7 (*n*=2)	F 5–5 (*n*=1)	F1–21 (*n*=1)	F 5–8 (*n*=1)	F1–1 (*n*=1)	F 3–6 (*n*=1)	F 3–7 (*n*=1)
	F 3–1 (*n*=1)	F 1–5 (*n*=1)						
	F1–112 (*n*=1)							
	nd (*n*=6)							
**PenA**	3 (*n*=8)	1 (*n*=2)	nd (*n*=1)	2 (*n*=1)	nd (*n*=1)	1 (*n*=1)	2 (*n*=1)	1 (*n*=1)
		9 (*n*=1)						

CC, clonal complex; MenB, C, Y and W, Meningococcal serogroup B, C, Y and W; nd, not determined; ST, sequence type.

For PorA (VR1, VR2 and VR3), the most common subtypes were P15 (*n*=7), P1.19 (*n*=7) and P36 (*n*=7). For PorB, the most common serotypes were 3–1 (*n*=11). The characterization of the PenA resistance gene in the 18 isolates was studied, the PenA3 allele was the most widespread (*n*=9).

## Discussion

In this work, the age group most affected by IMD is between 1 and 4 years old and represents 35 % of cases. Comparable results have been reported by several studies, in Algeria, more than 46 % of the cases affected the age group of less than 10 years old [12]. In addition, in Tunisia, IMDs mainly affect infants at 44.6 % [13]. In the USA, the incidence of the disease is higher in the age group between 1 and 4 years [14]. In France, the IMD notification rate is highest in infants younger than 1 year, followed by children aged 1–4 years [15].

Given that the age of predilection for invasive meningococcal infections is between 1 and 4 years of age, it is recommended that epidemiological surveillance of these infections be carried out in young children to avoid complications and serious sequelae. This situation has taken hold during the last few years, with several countries decimated.

Surveillance of confirmed IMD cases in Casablanca (Morocco) highlighted three important changes: an increase of invasive infection cases due to serogroup B strains with a prevalence ranging from 75.5 % [16] to 93.06 % cases; an increase in serogroup W, nine cases in our study which have been confirmed since 2010. Finally, we note the presence of three cases of serogroup Y with a first appearance in 2013 in the Casablanca region [17]. In the Maghreb region, Meningococcal serogroup B represents 80.4 % of cases in Tunisia [18], moreover, in Algeria it represents 46.7 % of cases between 2003 and 2013 [19]. Serogroup B predominates in Europe, North America and Australia [3]. In France, 66 % of the isolates are of serogroup B in 2017 [20], this figure rose to 52 % in 2018 [21]. An IMD surveillance study in Portugal showed that 41.8 % of cases were due to serogroup B [22]. In Spain, 71.2 % of IMDs are due to serogroup B [23].

Serogroup W was introduced to Morocco after the Haj period in 2000 when Saudi Arabia experienced outbreaks of meningococcal disease due to this serogroup. Since then, at national level, its prevalence has fluctuated between 2 and 30 % depending on the year [24]. Several countries have then reported cases of IMD due to serogroup W, which are linked with the travel to Saudi Arabia during the pilgrimage seasons (Hajj and Umrah) [25]. Serogroup W appears to have increased worldwide since 2013 [26]. In the Maghreb region, serogroup W represents in Tunisia a prevalence of 1.6 % [13] and 14 % in Algeria between 1992 and 2013 [19]. In France, 9 % of isolates are due to this serogroup [21]. In the Central African Republic, out of 80 samples tested, 66 belong to serogroup W [27].

During this period, only three cases (1.22%) of serogroup Y were confirmed in Casablanca. In Tunisia, the serogroup Y represents 4.7 % (*n*=59) [13]. During the period 2005–2016, in Mexico, serogroup Y represents 23.53 % [28]. In one region of Brazil in 2014, 8.5% cases are of serogroup Y [28]. In Japan, this serogroup was the most dominant with a percentage of 42 % at the end of 2014 [29]. In France, 12 % of the isolates are from serogroup Y [21]. A European study has shown a high proportion of meningococcal infections caused by serogroup Y, reaching more than 50 % in Sweden. In Switzerland, serogroup Y is the second cause of IMD, which represents 19 % [30].

Serogroup C represents 2.04 % of cases in Casablanca. Thus at the national level, 10 % of the isolates are of serogroup C [24]. This figure remains much lower than that of Tunisia where serogroup C represents 12.2 % [18], 8 % in Algeria [19], 26 % in France [21], 50 % in Portugal [22] and 61 % in Switzerland [31].

In Morocco, the predominance of serogroup B also dates back to the 1990s, when the first waves of meningococcal meningitis occurred in Morocco, namely the one between 1967–1968 and the other between 1988–1989 [24], with serogroup A predominating. This situation was similar to that of the meningitis belt during this period. Since that date, we have witnessed a progressive decrease in the prevalence of serogroup A [16], and an increase in serogroup B [17]. This epidemiological variation could be related to the proximity and trade with Europe and partly due to the vaccination of pilgrims (polysaccharide vaccine and ACXY conjugate vaccine).

Prompt antibiotic therapy is crucial to determining the outcome of meningococcal infection. Continuous monitoring is therefore necessary to monitor trends in antibiotic susceptibility in *N. meningitidis.*

The majority of the isolates in this study are trends in antibiotic susceptibility to penicillin G (72.6%). The proportion of *N. meningitidis* isolates with decreased susceptibility to penicillin G (MDSP) has shown an evolution since 1992. This MDSP rate passed from 4.3 % between 1992 and 2000 [16] to 8 % between 2003 and 2013 (IR-UHC bacteriology laboratory data) to 24 % between 2010 and 2019. The most part of MDSP, 37/44(84.09 %) belong to group B.

In Tunisia, the evolution of MDSP passed from 55.7 % combined in 2013 [18] to 81 % in 2016 [13]. In Algeria, MDSP represents 10.4 % between 1992 and 2013 [19].

In Portugal, 24.6 % of MDSP mainly associated with serogroup C (32.8%) [32]. In Belgium from 2000 to 2010, 14.1 % had MDSP [33]. In France, 31.7 % of MDSP were of serogroup C (40.6%) and serogroup W (37.6%) [34]. In England and Wales, the prevalence of isolates with decreased susceptibility to penicillin was higher in serogroup B isolates [34]. However in Argentina, the percentage of MDSP represents 35.4 % [35], 45 % in Italy [36], 57 % in Brazil [37], 67 % in Spain [38] and 21.7 % in Ontario, Canada [39].

No high-level resistance was observed concerning C3G. Similar results were found between 1992 and 2000 in Casablanca [16]. In France, 2 % of the strains isolated between 2012 and 2015 have a decreased susceptibility to C3G, whereas all the strains were sensitive before this date [37].

Regarding rifampicin, 33 isolates (18%) have a decreased susceptibility. In contrast, in Brazil 0.2 % of the meningococcal strains exhibited high resistance to rifampicin in 2008 [40]. In France, two cases of serogroup C that are resistant to rifampicin were reported in 2012 [41].

This phenomenon that has been observed in several countries around the world due to the acquisition of the mosaic genes. The surveillance of the state of decreased susceptibility to penicillin G remains a marker for the epidemiological monitoring of meningococcal. In addition, one should not forget the other antibiotics used either in therapeutic treatment or prophylaxis. The dynamic changes in the distribution of *N. meningitidis* serogroups and the risk of increasing decreased susceptibility to antibiotics used in treatment and prophylaxis justify ongoing surveillance of these isolates.

MLST analysis of 14 strains of meningococcus B among all the strains isolated from this serogroup showed a major predominance of the clonal complex CC 32(9/14). A previous study conducted in Casablanca showed that the majority of the strains belonged to this CC 32 complex [42]. In addition, in Tunisia, CC 32 represents only 9.7 % of isolates [18]. In France, 26 % of the isolates belonged to CC 32 between 2006 and 2015 [20]. In Brazil, 72 % of the meningococcal strains belong to CC 32 [43]. In Spain, 95.83 % of the cases belong to CC 32, it is therefore a globally frequent hyper-invasive clone [44].

The MLST strains analysed in this study (particularly for serogroup B isolated from children) were predominantly CC 32. This can be explained by frequent genetic modifications that are probably due to recombinations. This mechanism is predominant in genetic variations of meningococcal serogroup B [45, 46].

## Conclusion

IMDs are notifiable in Morocco. These infections by their seriousness and their transmissibility require immediate diagnosis, therapeutic treatment and suitable prophylaxis. The rate of strains of reduced susceptibility to penicillin G has increased. In Grand Casablanca, despite a decrease in cases of meningococcal meningitis, most IMDs are due to serogroup B. Regular and continuous epidemiological surveillance is essential for controlling the appearance or increase of invasive clones.

## References

[1] Harrison LH, Pelton SI, Wilder-Smith A, Holst J, Safadi MAP (2011). The global meningococcal initiative: recommendations for reducing the global burden of meningococcal disease. Vaccine.

[2] Agnememel A, Hong E, Giorgini D, Nuñez-Samudio V, Deghmane A-E (2016). Neisseria meningitidis serogroup X in sub-Saharan Africa. Emerg Infect Dis.

[3] Borrow R, Caugant DA, Ceyhan M, Christensen H, Dinleyici EC (2017). Meningococcal disease in the middle East and Africa: findings and updates from the global meningococcal initiative. J Infect.

[4] Jones DM, Sutcliffe EM (1990). Meningococci with reduced susceptibility to penicillin. Lancet.

[5] Nadel S (2016). Treatment of meningococcal disease. J Adolesc Health.

[6] Rouphael NG, Stephens DS (2012). Neisseria meningitidis: biology, microbiology, and epidemiology. Methods Mol Biol.

[7] Borrow R, Alarcón P, Carlos J, Caugant DA, Christensen H (2017). The global meningococcal initiative: global epidemiology, the impact of vaccines on meningococcal disease and the importance of herd protection. Expert Rev Vaccines.

[8] (2001). M100-S11, performance standards for antimicrobial susceptibility testing. Clin Microbiol Newsl.

[9] Taha MK (2000). Simultaneous approach for nonculture PCR-based identification and serogroup prediction of Neisseria meningitidis. J Clin Microbiol.

[10] Maiden MC, Bygraves JA, Feil E, Morelli G, Russell JE (1998). Multilocus sequence typing: a portable approach to the identification of clones within populations of pathogenic microorganisms. Proc Natl Acad Sci U S A.

[11] Vogel U, Taha M-K, Vazquez JA, Findlow J, Claus H (2013). Predicted strain coverage of a meningococcal multicomponent vaccine (4CMenB) in Europe: a qualitative and quantitative assessment. Lancet Infect Dis.

[12] Tali-Maamar H, Rahal K (2003). Étude de souches de Neisseria meningitidis isolées en Algérie entre 1992 et 2001. Médecine et Maladies Infectieuses.

[13] Brik A, Terrade A, Hong E, Deghmane A, Taha MK (2020). Phenotypic and genotypic characterization of meningococcal isolates in Tunis, Tunisia: high diversity and impact on vaccination strategies. Int J Infect Dis.

[14] Baccarini C, Ternouth A, Wieffer H, Vyse A (2013). The changing epidemiology of meningococcal disease in North America 1945-2010. Hum Vaccin Immunother.

[15] Thabuis A, Tararbit K, Taha M-K, Dejour-Salamanca D, Ronin V (2018). Community outbreak of serogroup B invasive meningococcal disease in Beaujolais, France, February to June 2016: from alert to targeted vaccination. Eurosurveillance.

[16] Zerouali K, Elmdaghri N, Boudouma M, Benbachir M, Serogroups BM (2002). Serogroups, serotypes, serosubtypes and antimicrobial susceptibility of Neisseria meningitidis isolates in Casablanca, Morocco. Eur J Clin Microbiol Infect Dis.

[17] Razki A, Hong E, Zerouali K, Belabbes H, Aitmouss K (2018). Molecular characterization of invasive isolates of Neisseria meningitidis in Casablanca, Morocco. J Clin Microbiol.

[18] Saguer A, Smaoui H, Taha M-K, Kechrid A (2016). Characterization of invasive Neisseria meningitidis strains isolated at the children's Hospital of Tunis, Tunisia. East Mediterr Health J.

[19] Maamar HT, Laliam R, Guettou B, Rahal K (2017). Molecular typing and antibiotic sensitivity of Neisseria meningitidis strains isolates in Algeria.

[20] Parent du Chatelet I, Deghmane AE, Antona D, Hong E, Fonteneau L (2017). Characteristics and changes in invasive meningococcal disease epidemiology in France, 2006-2015. J Infect.

[21] Hong E, Barret A-S, Terrade A, Denizon M, Antona D (2018). Clonal replacement and expansion among invasive meningococcal isolates of serogroup W in France. J Infect.

[22] Caniça M, Dias R, Nunes B, Carvalho L, Ferreira E (2004). Invasive culture-confirmed Neisseria meningitidis in Portugal: evaluation of serogroups in relation to different variables and antimicrobial susceptibility (2000–2001). J Med Microbiol.

[23] Garrido-Estepa M, Guzman M, Portero R (2014). Enfermedad meningocócica en España. Análisis de la temporada 2012–2013.

[24] Ministère de la Santé, Maroc Guide de la lutte contre les méningites bactériennes communautaires, Juin 2010. www.sante.gov.ma.

[25] Memish ZA, Shibl AM (2011). Consensus building and recommendations based on the available epidemiology of meningococcal disease in Gulf cooperation Council states. Travel Med Infect Dis.

[26] Lucidarme J, Hill DMC, Bratcher HB, Gray SJ, du Plessis M (2015). Genomic resolution of an aggressive, widespread, diverse and expanding meningococcal serogroup B, C and W lineage. J Infect.

[27] Frank T, Hong E, Mbecko J-R, Lombart J-P, Taha M-K (2018). Emergence of Neisseria meningitidis Serogroup W, Central African Republic, 2015–2016. Emerg Infect Dis.

[28] Vespa Presa J, Abalos MG, Sini de Almeida R, Cane A (2019). Epidemiological burden of meningococcal disease in Latin America: a systematic literature review. Int J Infect Dis.

[29] Fukusumi M, Kamiya H, Takahashi H, Kanai M, Hachisu Y (2016). National surveillance for meningococcal disease in Japan, 1999–2014. Vaccine.

[30] Bröker M, Emonet S, Fazio C, Jacobsson S, Koliou M (2015). Meningococcal serogroup Y disease in Europe: continuation of high importance in some European regions in 2013. Hum Vaccin Immunother.

[31] Ruedin HJ, Ninet B, Pagano E, Rohner P (2004). Epidemiology of meningococcal disease in Switzerland, 1999–2002. Eur J Clin Microbiol Infect Dis.

[32] Ferreira E, Dias R, Caniça M (2006). Antimicrobial susceptibility, serotype and genotype distribution of meningococci in Portugal, 2001-2002. Epidemiol Infect.

[33] Bertrand S, Carion F, Wintjens R, Mathys V, Vanhoof R (2012). Evolutionary changes in antimicrobial resistance of invasive Neisseria meningitidis isolates in Belgium from 2000 to 2010: increasing prevalence of penicillin nonsusceptibility. Antimicrob Agents Chemother.

[34] Antignac A, Ducos-Galand M, Guiyoule A, Pirès R, Alonso J-M (2003). Neisseria meningitidis strains isolated from invasive infections in France (1999–2002): phenotypes and antibiotic susceptibility patterns. Clin Infect Dis.

[35] Sorhouet-Pereira C, Efron A, Gagetti P, Faccone D, Regueira M (2013). Phenotypic and genotypic characteristics of Neisseria meningitidis disease-causing strains in Argentina, 2010. PLoS One.

[36] Vacca P, Fazio C, Neri A, Ambrosio L, Palmieri A (2018). *Neisseria meningitidis* Antimicrobial Resistance in Italy, 2006 to 2016. Antimicrob Agents Chemother.

[37] Gorla MC, Pinhata JMW, Dias UJ, de Moraes C, Lemos AP (2018). Surveillance of antimicrobial resistance in Neisseria meningitidis strains isolated from invasive cases in Brazil from 2009 to 2016. J Med Microbiol.

[38] Pascual A, Joyanes P, Martinez-Martinez L, Suarez AI, Perea EJ (1996). Comparison of broth microdilution and E-test for susceptibility testing of Neisseria meningitidis. J Clin Microbiol.

[39] Brown EM, Fisman DN, Drews SJ, Dolman S, Rawte P (2010). Epidemiology of invasive meningococcal disease with decreased susceptibility to penicillin in Ontario, Canada, 2000 to 2006. Antimicrob Agents Chemother.

[40] Gorla MCO, de Paiva MV, Salgueiro VC, Lemos APS, Brandão AP (2011). Antimicrobial susceptibility of Neisseria meningitidis strains isolated from meningitis cases in Brazil from 2006 to 2008. Enferm Infecc Microbiol Clin.

[41] Mounchetrou Njoya I, Deghmane A, Taha M, Isnard H, Parent du Chatelet I (2012). A cluster of meningococcal disease caused by rifampicin-resistant C meningococci in France, April 2012. Euro Surveill.

[42] Zerouali K, Castelli P, Van Looveren M, El Mdaghri N, Boudouma M (2006). Étude de souches de Neisseria meningitidis sérogroupe B isolées à Casablanca PAR multilocus sequence typing et électrophorèse en CHAMP pulsé. Pathol Biol.

[43] Silva LA, Coronato B, Schlackman J, Marsh JW, Ezeonwuka C (2018). *Neisseria meningitidis* disease-associated clones in Amazonas State, Brazil. Infect Dis.

[44] Abad R, Medina V, Stella M, Boccadifuoco G, Comanducci M (2016). Predicted strain coverage of a new meningococcal multicomponent vaccine (4CMenB) in Spain: analysis of the differences with other European countries. PLoS One.

[45] Feil EJ, Maiden MC, Achtman M, Spratt BG (1999). The relative contributions of recombination and mutation to the divergence of clones of Neisseria meningitidis. Mol Biol Evol.

[46] Jolley KA, Kalmusova J, Feil EJ, Gupta S, Musilek M (2000). Carried meningococci in the Czech Republic: a diverse recombining population. J Clin Microbiol.

